# Phenotypic and Genomic Analysis of Cystic Hygroma in Pigs

**DOI:** 10.3390/genes12020207

**Published:** 2021-01-31

**Authors:** Anna Letko, Alexandria Marie Schauer, Martijn F. L. Derks, Llorenç Grau-Roma, Cord Drögemüller, Alexander Grahofer

**Affiliations:** 1 Institute of Genetics, Vetsuisse Faculty, University of Bern, 3012 Bern, Switzerland; anna.letko@vetsuisse.unibe.ch (A.L.); cord.droegemueller@vetsuisse.unibe.ch (C.D.); 2 Institute of Animal Pathology, Vetsuisse Faculty, University of Bern, 3012 Bern, Switzerland; alexandria.schauer@vetsuisse.unibe.ch (A.M.S.); llorenc.grauroma@vetsuisse.unibe.ch (L.G.-R.); 3 Topigs Norsvin Research Center, 6640 AA Beuningen, The Netherlands; martijn.derks@topigsnorsvin.com; 4 Animal Breeding and Genomics Group, Wageningen University, 6700 Wageningen, The Netherlands; 5 Clinic for Swine, Vetsuisse Faculty, University of Bern, 3012 Bern, Switzerland

**Keywords:** *Sus scrofa*, precision medicine, lymphatic system, whole-genome sequencing, SNP array genotyping

## Abstract

Cystic hygroma is a malformation of the lymphatic and vascular system and is recognized as a benign congenital tumor that affects humans and animals in the perinatal period. This congeni-tal disorder is rarely described in animals, and until today, cystic hygroma in pigs has not been described in the literature. In a purebred Piètrain litter with twelve live-born piglets, cystic hy-groma was noticed on the rump of two male pigs within the first week of life. In addition, a third case of a crossbred weaner (Large White × Landrace) was detected during a herd examina-tion. To rule out common differential diagnoses, e.g., abscess or hematoma, further clinical and pathological investigations were conducted. During clinical examination, a painless and soft mass, which was compressible, was detected on the rump of all affected animals. The ultra-sonographic examination revealed a fluid-filled and cavernous subcutaneous structure. In addi-tion, a puncture of the cyst was conducted, revealing a serosanguinous fluid with negative bacte-riological culture. In all cases, a necropsy was performed, showing that the animals had fluid-filled cysts lined by well-differentiated lymphatic endothelium. Based on the clinicopathological examination, cystic hygroma was diagnosed. Furthermore, SNP array genotyping and whole-genome sequencing was performed and provided no evidence for a chromosomal disorder. In the Piètrain family, several genome regions were homozygous in both affected piglets. None-theless, a dominant acting de novo germline variant could not be ruled out, and therefore differ-ent filtering strategies were used to find pathogenic variants. The herein presented lists of pri-vate variants after filtering against hundreds of control genomes provide no plausible candidate and no shared variants among the two sequenced cases. Therefore, further studies are needed to evaluate possible genetic etiology. In general, systematic surveillance is needed to identify ge-netic defects as early as possible and to avoid the occurrence of losses in the pig population.

## 1. Introduction

Cystic hygroma, often also referred to as ‘cystic lymphangioma’, is one of the most commonly presenting lymphangioma in human medicine. It is a well-known congenital malformation of the lymphatic system characterized as single or multiloculated fluid-filled cavities due to a lack of communication between the lymphatic and venous systems [1,2,3,4]. Cystic hygroma occurs with an incidence of ~1:1000–6000 births and 1:750 miscarriages in humans [5,6]. Even though cases of cystic hygroma are rarely described in animals [7,8,9,10,11], an estimation of the prevalence of this malformation in animals is still missing. Cystic hygromas can manifest anywhere in the body but are often found in the neck, clavicle, and axillary regions in humans [1,2,3,4,12]. In approximately half of the reported cases, cystic hygromas are present directly after birth, whereas the other cases occur within the age of two years [13]. Until today, the exact etiology of cystic hygroma in humans and animals has been unclear, but an association with chromosomal aberrations and genetic syndromes, such as the Noonan syndrome (OMIM PS163950), has been described [1,2,3,12]. Abnormal karyotype was found in 29% to 60% of the cases [14], whereas congenital disorders with normal karyotype ranged from 25% to 53% [15]. However, submicroscopic chromosomal abnormalities that are missed by conventional karyotyping are also described in cystic hygroma [2]. In addition, cases of familial cystic hygroma with normal karyotype have been described and suggest that both recessively as well as dominantly inherited genetic variants are involved in the phenotype [2,16,17,18,19].

Until now, no information regarding the occurrence, the etiology, and the genetic background of cystic hygroma in the pig population has been available. The clinical phenotype of cystic hygroma in pigs resembles the human condition as well as reports of similarly affected individuals of other domestic animal species. To the authors’ knowledge, this is the first report of the hygroma cyst in pigs. Therefore, this report describes the phenotypic findings of hygroma cysts in three pigs of different breeds and the subsequent preliminary genomic analysis, including SNP genotyping and whole-genome sequencing, to evaluate a possible inherited cause.

## 2. Materials and Methods

### 2.1. Ethics Statement

All animal experiments were performed according to the local regulations. The study was approved by the Cantonal Committee for Animal Experiments (Canton of Bern; permit 109/18) at the University of Bern.

### 2.2. Animals and DNA Samples

Three male cases with cystic hygroma on the rump were used in this study. Two cases were littermates from a purebred Piètrain litter. The third case, a crossbred weaner (Large White × Landrace), was observed during a herd examination in a fattening herd. Blood samples were obtained from all cases as well as from the Piètrain sow, boar, and all 20 healthy siblings from two independent litters after repeated mating for further genetic investigations. Genomic DNA was isolated from EDTA blood samples using the Maxwell RSC Whole Blood DNA Kit (Promega AG, Dübendorf, Switzerland).

### 2.3. Clinical and Further Examination

A total of three male cases with cystic hygroma on the rump were examined in this study. Two cases (case 1 and 2) were littermates from a purebred Piètrain litter born at the Clinic for Swine in Bern. The pregnant Piètrain sow, artificially inseminated from a Piètrain boar of a boar study, was bought from a nucleus farm in Switzerland and farrowed at the Clinic for Swine. The sow was raised under conventional conditions. At the beginning of gestation, the sow was kept in a group house system with straw as a bedding material according to legal requirements and received a conventional feed diet and water ad libitum. At the Clinic for Swine, the animal was housed in a single pen with contact to other pigs. The pen was interspersed with straw and sawdust. In addition to the conventional feed, the sow also received hay. Within the first week after farrowing, cystic hygroma occurred in two (male) out of eleven (3 female, 8 male) piglets. A rebreeding of the sire and dam under experimental conditions was conducted to investigate a possible genetic effect. The litter size of the second litter was twelve (7 female, 5 male), but no more affected piglets were observed during the lactation period of four weeks. Due to leg weakness, no further breeding with the sow was possible, and therefore, no further rebreeding was conducted.

The third case (case 3) was observed during a herd examination in a fattening herd. The farmer reported that this weaner (Large White × Landrace, male castrated) already arrived with the cystic hygroma to his farm from a piglet producer. No information about the mother and father could be obtained. However, this animal was referred to the Clinic for Swine for further investigation. A clinical examination was conducted in all affected animals. Further investigations, including ultrasonography and cytology of the fluid in the cyst, were conducted to clarify the phenotype of the cystic hygroma and rule out differential diagnoses.

### 2.4. Postmortem Examination, Histology, and Bacteriology

A full postmortem was performed immediately after euthanasia on the two affected Piètrain littermates (cases 1 and 2), and one crossbred weaner pig (case 3). Tissue samples from the skin, subcutaneous lesions, skeletal muscle, superficial inguinal lymph nodes, and internal organs including lung, liver, and kidney from all pigs and from a mass observed within the radius in case 1 were fixed in 10% buffered formalin, pH 7.2, overnight. All tissues were routinely processed for histopathology and stained with hematoxylin and eosin (H&E). Immunohistochemical examination was performed using rabbit polyclonal antibodies against LYVE1 (Abcam, Cambridge, UK), at the dilution of 1:3000, and counterstained with hematoxylin. Inguinal lymph nodes were included as an internal positive control.

Sterile samples for bacteriological investigations of the fluid present within the cystic masses were collected before sectioning the tissues in all three cases. The fluid samples were cultured aerobically on blood agar with and without ammonia and on MacConkey agar for 48 h. As part of the national surveillance system, blood samples were tested for the presence of antibodies against African swine fever virus (ASFV), Classical swine fever virus (CSFV), and Porcine reproductive and respiratory syndrome virus (PRRSV) by ELISA.

### 2.5. Genetic Analyses

From both of the purebred Piètrain litters, 24 animals (dam, sire, 2 affected piglets, and 20 apparently normal littermates) were genotyped using the Illumina PorcineSNP60 BeadChip (Illumina, San Diego, CA, USA) containing 50,915 SNPs. Basic quality control filtering steps of the SNP array genotyping data and parentage confirmation were carried out using PLINK v1.9 [20]. Markers with call rates <90% were excluded, and all individuals had call rates >90%. The dataset was additionally pruned for low minor allele frequency (0.05) and failure to meet Hardy–Weinberg equilibrium (0.0001), resulting in 31,054 markers. The dataset was also scanned for Mendelian errors using the --mendel option of PLINK to reveal any deviations from expected values based on per-individual, per-family, and per-SNP error rates.

Merlin software [21] was used to test for cosegregation of any chromosomal regions and the cystic hygroma phenotype in one complete family representing the first litter (sire, dam, two affected, and eight unaffected offspring) by performing parametric linkage analysis under a fully penetrant, recessive model of inheritance. Assuming identity-by-descent (IBD), the autozygosity mapping approach in PLINK v1.9 [20] was used to discover homozygous intervals with alleles shared by both affected piglets (using --homozyg-match 0.95 for allelic matching between both cases). Additionally, individual homozygous intervals were determined in the 22 control animals for comparison. All plots were constructed in R environment v3.6.0 [22].

### 2.6. Whole-Genome Sequencing

Whole-genome sequence (WGS) data were obtained from five pigs including four animals of the purebred Piètrain litter (case 1, its dam, sire, and one normal littermate), as well as the unrelated affected crossbred pig (case 3), after preparation of PCR-free fragment libraries with approximately 400 bp inserts that were sequenced for paired-end reads of 2 × 150 bp length. All five animals were sequenced on the Illumina NovaSeq 6000 System at an average coverage of 21× (Supplementary Table S1). The obtained reads were mapped to the pig reference genome assembly Sscrofa11.1 using the Burrows-Wheeler Aligner v0.7.15 [23] with default settings. Picard v2.9 [24] was used to sort the mapped reads by the sequence coordinates and to label the read duplicates. Genome Analysis Toolkit v3.8 (GATK) [25] was used to perform local realignment and to produce a cleaned BAM file. The single nucleotide variants (SNVs) and small indels were identified using genotypeGVCFs of GATK, and the prediction of their functional effects was performed with SnpEff v4.3 [26] using the NCBI Annotation Release 106. The generated files were merged with 10 other unrelated pig genomes (Supplementary Table S1), which were generated previously during the course of different studies and are publicly available, into a final variant call format (VCF) file, including all individual variants and their functional annotations. The VCF file was then used for determining private variants of the affected animals.

As an independent validation, the obtained variants were additionally searched in a cohort of 756 commercial pig genomes from four breeds (Duroc, Large White, Landrace, Piètrain). This cohort of sequenced animals was analyzed according to Derks et al., 2019 [27]. In short, sequence reads were mapped using Burrows–Wheeler aligner against the Sscrofa11.1 reference genome, and SAMtools [23] was used to sort, merge, and index BAM files. We performed variant calling using FreeBayes with the setting: --min-base-quality 10 --min-alternate-fraction 0.2 --haplotype-length 0 --min-alternate-count 2 [28]. We discarded variants with a Phred quality <20. The resulting sequence variants were functionally annotated using the Ensembl Variant Effect Predictor pipeline (v99) [29].

The Integrative Genomics Viewer (IGV) [30] was used for visual inspection and screening for structural variants in the regions of interest in the WGS of the affected pigs. Additionally, coverage for the five animals was calculated using the function bedcov of the program Samtools [23] by a sliding window approach with the window size of 300 kb moving for half the window size and including reads with mapping quality greater than 15. The average coverage over every window was plotted for each pig with the qqman package [31] in R environment v3.6.0 [22].

### 2.7. Availability of Data and Material

All positions refer to the pig reference genome assembly Sscrofa11.1 and NCBI Annotation Release 106. The herein generated WGS data are freely available at the European Nucleotide Archive (ENA) under study accession number PRJEB29465, and individual sample accession numbers are available in Supplementary Table S1. The genotypes of the commercial pig genomes used as the validation cohort within the candidate regions are available on reasonable request.

## 3. Results

### 3.1. Clinical and Further Examination

**Figure 1:**
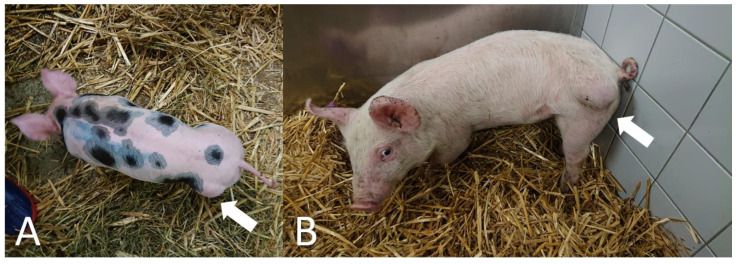
Phenotypic appearance of cystic hygroma in pigs. A soft and compressible mass on the rump of two cases is visible ((**A**) = case 1; (**B**) = case 2).

**Figure 2:**
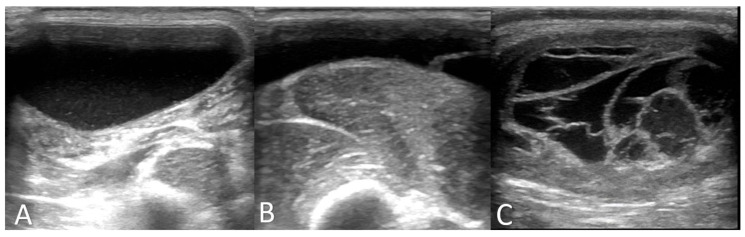
Ultrasonographic imaging of cystic hygroma in pigs. Fluid-filled cysts ((**A**) = case 1; (**B**) = case 2; (**C**) = case 3) with cavernous structure (**C**) could be detected.

A clinical examination of all three cases revealed that the animals were alert and in good body condition. The vital signs were within the reference values, and no locomotion or neurological disorders were determined. In all three animals, a painless, soft, and compressible mass on the rump was detected (Figure 1, Supplementary Video S1, and Supplementary Video S2); and an ultrasonographic examination revealed a cystic or multicystic lesion with internal septations (Figure 2). In addition, after sterile preparation, an aspiration of the fluid was conducted in all three animals and a cytological investigation was performed. Results from the cytological examination are listed in Table 1.

Based on the findings of the clinical examination, the diagnosis of cystic hygroma was made in all three affected pigs. In addition, a clinical examination of all controls revealed that the animals were alert and in good body condition. The vital parameters were all within the reference range, and examination of the locomotion and neurological system revealed no pathological findings. Based on the results of the clinical examination, all controls were healthy.

### 3.2. Postmortem Examination, Histology, and Bacteriology

**Figure 3:**
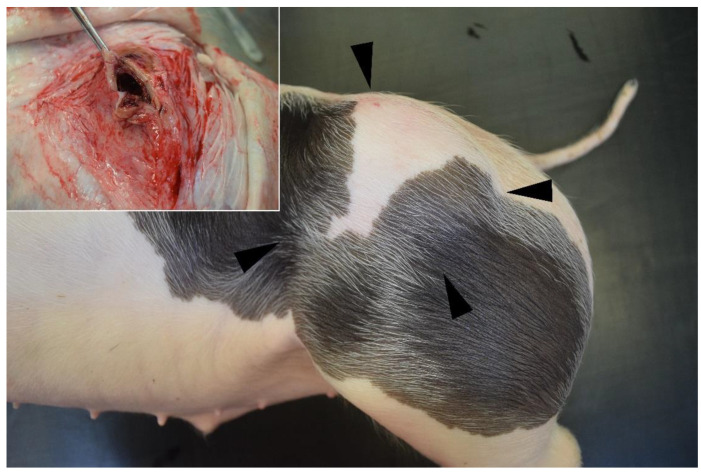
Porcine cystic hygroma (case 1). Note the well-demarcated, subcutaneous mass located at the level of the left rump (arrowheads). Inset: The mass is surrounded by a thick, fibrous capsule and contains a central cystic cavity filled with serosanguineous fluid.

The gross and histological appearance of the masses was similar in all three pigs. The masses were grossly palpable, fluctuant, well-demarcated, and located subcutaneously at the level of the rump. The masses were 9 × 7 × 3 cm, 10 × 8 × 4 cm, and 17 × 7 × 2 cm in cases 1 to 3, respectively. In the cut sections, the masses were surrounded by a thick, fibrous capsule with a width of 0.3–0.5 cm and contained multiple cystic cavities filled with serosanguineous fluid and a few aggregates of coagulated protein (Figure 3).

**Figure 4:**
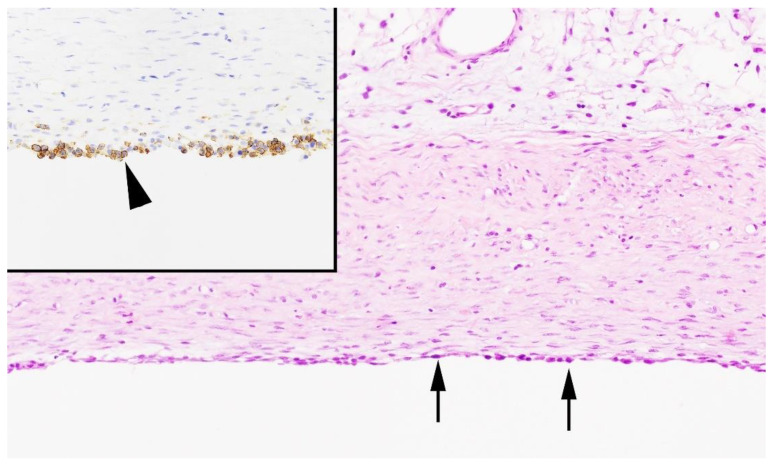
Histological phenotype of porcine cystic hygroma (case 1). Detail of the subcutaneous cystic mass surrounded by a thick capsule of mature connective tissue and lined by a single layer of well-differentiated cells with a squamous to cuboidal morphology (arrows). Inset: Immunohistochemistry for LYVE-1. Most of the epithelial cells lining the cystic cavity are diffusely and strongly positively stained with hematoxylin and eosin (arrowhead).

Histologically, the masses were limited to the subcutis, and the cystic cavities were surrounded by a thick capsule comprised of mature fibrous connective tissue. The innermost layer of the capsule was lined by a single layer of cells that were mostly flat but multifocally plump to cuboidal. Cells contained a moderate amount of pale, eosinophilic cytoplasm and one central, round to ovoid nucleus. The central cavity was filled with pale, extracellular eosinophilic material, a low amount of fibrin, and a few scattered neutrophils and lymphocytes. Immunohistochemistry for LYVE-1 demonstrated diffuse and strongly positive staining within the cells lining the cystic masses, which was consistent with lymphatic endothelium (Figure 4). The endothelial cells lining the afferent and efferent lymphatic vessels of the lymph node as well as the subcapsular and peritrabecular sinuses showed similar positive staining, indicating the success of the positive control.

No other relevant lesions were observed in the three affected pigs. No bacterial growth was obtained in the bacteriological investigations of any of the collected samples. The serology for ASFV, CSFV, and PRRSV was negative.

### 3.3. Genetic Analyses

A total of 24 pigs from two Piètrain litters were genotyped using the SNP array. The same parentage of both litters was confirmed by the IBD estimates for all pairs of individuals. No increased number of Mendelian errors between the parents and the two affected offspring (case 1 and case 2) was observed, indicating no larger structural variants in the genome of the affected piglets.

Based on the pedigree structure, initially, an autosomal recessive inheritance was suspected. Parametric linkage analysis for a recessive trait in the first litter resulted in ten genome regions with a positive logarithm of the odds (LOD) score (Supplementary Table S2). Moreover, ten runs of homozygosity shared by the two Piètrain cases were detected on nine autosomes (chr 1, 3, 5, 6, 11, 12, 14, 17, and 18). Only one homozygous segment overlapped with a linked interval on chromosome 12, forming a ~4.6 Mb (chr 12: 178,275–4,826,688) region of interest (Supplementary Table S2). However, from 9% to 100% of the 22 control pigs were also homozygous over the ten detected homozygous regions (Supplementary Figure S1, Supplementary Table S2).

As both affected piglets of the Piètrain litter were males, an X-linked recessive inheritance could also be assumed. SNP genotyping showed that both cases received three IBD segments on chromosome X (Supplementary Table S2). Subsequently, we noticed that these haplotypes also occurred in several unaffected littermates as in these three segments, the unaffected male siblings received the identical maternal X chromosomes (Supplementary Table S3).

### 3.4. Whole-Genome Sequencing

The genomes of one affected Piètrain piglet (case 1), both parents, one healthy littermate, and the unrelated case 3 were sequenced. All five cleaned BAM files were inspected for coverage differences, and no evidence for chromosomal imbalance was detected (Supplementary Figure S2).

Subsequently, we focused genome-wide on variants in annotated genes and loci, assuming a protein-changing variant was causing the observed phenotype. For the sequenced Piètrain family consisting of four animals, we applied three different strategies to filter for disease-associated variants assuming various modes of inheritance, including autosomal recessive (AR), X-linked recessive (XR), and trio filtering for possible autosomal dominant (AD) de novo variants (Table 2). For the crossbred case 3, we searched for private heterozygous and homozygous variants that were absent in all other available genomes (Table 2).

Filtering for SNVs and small indels was performed against 10 unrelated control pigs (Supplementary Table S1) and revealed 104 coding variants detected in the purebred Piètrain case 1 based on different filtering strategies (Table 2, Supplementary Table S4). After a second round of filtering using the independent validation cohort of 756 commercial pig genomes, 22 coding variants of which 8 were predicted to be protein-changing remained. These were present only in the genome of the affected piglet from the Piètrain litter and absent in all controls (Table 2). In light of the outcome of the previously performed genetic analyses, only a single private protein-changing variant in *LOC110255918* mapped to the IBD genome region on chromosome 12 (Supplementary Table S4).

In the WGS data of case 3, we found 2585 variants; after the second round of filtering, this list was reduced to 500 private SNVs and small indels, including 2 nonsense, 6 frameshift, 184 missense, 216 synonymous, and 86 intronic variants, as well as 3 inframe insertions, 2 inframe deletions, and 1 bidirectional gene fusion (Table 2, Supplementary Table S5).

No private variants shared between the two sequenced affected pigs were found. Two genes located on chromosome X were shared in case 1 and case 3, harboring independent private coding variants. The two different variants found in the chloride voltage-gated channel 4 (*CLCN4*) gene located in a homozygous region (Supplementary Table S4) were synonymous in both sequenced cases. In addition, the two different variants in the myotubularin related protein 1 (*MTMR1*) gene located in a linked region (Supplementary Table S4) were silent in case 1 and only predicted to alter the protein sequence in case 3.

## 4. Discussion

This is the first case report of cystic hygroma in pigs of different breeds in combination with further downstream analyses, including a comprehensive but still preliminary genomic analysis based on short-read whole-genome sequencing. Hence, a novel congenital disorder in pigs was described. In comparison with similar cases described in humans showing cystic hygroma in the neck region [1,2,3,4,12], in all three herein described porcine cases, the cystic hygroma was located at the level of the hind limb. Interestingly, no breathing problems, which are described in infants, could be observed in the pigs because the cystic hygroma was only located on the hind limb. However, all the pigs showed a higher risk of injuries on the rump, and therefore, this congenital disorder has implications for animal welfare. Moreover, the cystic hygroma on the limb tremendously influenced the meat quality, especially the dry ham production [32]. If the incidence of cystic hygroma in the pig population increased due to the high use of carrier boars, it would have a significant impact on the pig industry economy. Therefore, the results of this preliminary study are of major importance.

Due to the high use of semen from boar studs for artificial insemination, there is an increased risk of distributing a hereditary disease in the pig population [33,34,35]. Therefore, a thorough diagnostic and rapid analysis of this genetic disorder was conducted to avoid spreading of the disease in the pig population. The outcome of genetic analysis depends on the number of cases with a confirmed phenotype and the causative genetic variant. Hence, the diagnosis of the phenotype is of major importance, although systematic surveillance in the pig production is mostly lacking. In this study, all affected animals were confirmed by clinical examination and further pathological investigations to receive a valid phenotype for the subsequent genomic analysis. The clinical examination revealed all typical signs for hygroma cysts, including a painless, compressible, and soft mass on the rump [1,2]. To confirm this diagnosis, further investigations, including ultrasonographic examination, aspiration of the mass, and pathological examination were conducted. Findings showed cystic or multicystic lesions with internal septations and cloudy, reddish fluid with a low content of proteins, which was in line with the diagnosis of cystic hygroma [1,2]. Although half of the cases in human medicine are described at birth [13], all three herein described porcine cases developed the malformation within several days after birth, indicating a congenital condition.

As a familial occurrence of cystic hygroma is hypothesized in human medicine [16,17,18], we hypothesized a possible genetic origin for the observed cases in pigs. As the mode of inheritance was unclear, we evaluated different possible scenarios, such as monogenic recessive, X-linked, and dominant inheritance. As all three cases were male, a sex-linked inheritance seemed to be likely, and therefore, a second litter of the Piètrain sow with the same boar was produced under experimental conditions but revealed no further affected piglets. As all piglets of the second litter were apparently showing no signs of cystic hygroma until weaning, it might be speculated that due to the low number of male offspring, no further cases occurred. However, a resolution of the malformation on the lymphatic tissue has been proven by ultrasonographic examination during pregnancy [17]. This could not be ruled out in this study because the examination of all piglets in utero with ultrasonography is not possible in sows.

The outcome of the genetic and genomic analyses were inconclusive. There was at least a single genome region linked to the phenotype and showing shared homozygosity within the Piètrain family, and a single private protein-changing variant was found in that region on chromosome 12. Additionally, the condition may not be fully penetrant as the possibility that two normal littermates were identically homozygous for that genome region could not be ruled out. Using MutPred2 [36], the silico prediction of possible deleterious consequences for this missense variant in LOC110255918 revealed a score of 0.046, indicating no pathogenic effect. Nonetheless, the impact of this missense mutation in the uncharacterized locus remains unclear.

Furthermore, we opted for a possible sex-linked inheritance approach, which showed three shared IBD regions on chromosome X. However, the unaffected male littermates also shared these haplotypes. Only a single normal littermate shared the identical haplotypes in all three regions of the X chromosome, indicating that this offspring received the identical nonrecombinant copy of the maternal chromosome. Nonetheless, several further normal piglets received recombinant versions of the maternal X chromosome in the three distant chromosomal segments, resulting in several normal littermates sharing the respective haplotypes seen in both affected piglets. Therefore, the X-recessive mode of inheritance seems to be less likely. Furthermore, we could not rule out a possible paternal mosaicism of the X chromosome as an explanation. Filtering in the three regions revealed a variant in the CLCN4 gene that is known to be associated with the X-linked dominant inherited Raynaud-Claes syndrome, a very rare neurodevelopmental disease in humans (OMIM 300114). In addition to the fact that this synonymous variant does not alter the encoded protein, we postulate that the variant also found in the dam of the cystic hygroma-affected Piètrain piglet is most likely not causative. Indeed, sequencing the genome of the second affected littermate would have been beneficial in the identification of a possible shared causal variant. Recently it was evaluated that sequencing of each additional family member helped to narrow down the number of variants by 50%–75% [37]

In addition, the sequence analysis performed for the independent crossbred pig revealed no plausible candidate variant for the observed cystic hygroma phenotype that was highly similar to the two Piètrain cases. The private missense variant in MTMR1 located on the X chromosome seems to be less likely causative as the closely related MTM1 gene is known to be associated with forms of X-linked inherited myopathies (OMIM 300415). Furthermore, the long list of remaining private variants after filtering against hundreds of control genomes clearly illustrates that without sequencing of close relatives, ideally parents, as done for the Piètrain case, it is nearly impossible to limit the number of relevant variants if no obvious candidate gene is known. Furthermore, the chosen short-read genome sequencing method is known to be limited for the detection of structural variants. Therefore, long-read sequencing might be interesting in future cases to identify these kinds of genetic variations. Finally, the generated genome data revealed no indication of the presence of submicroscopic chromosomal abnormalities that were described in human cystic hygroma.

## 5. Conclusions

For the first time, this report provides a comprehensive description of cystic hygroma in pigs, including a preliminary genomic evaluation of possible inherited causes. It could be assumed that the genetic origin is heterogeneous as no shared variants across the two whole-genome sequenced affected pigs are found. Further targeted matings might help to elucidate the mode of inheritance. Systematic surveillance is needed to identify congenital defects as early as possible and to avoid the occurrence of further losses in the pig population.

## Figures and Tables

**Table 1:** Cytological examination.

Parameters	Case 1	Case 2	Case 3
**Colour**	Light red	Red	Red
**Transparency**	Cloudy	Cloudy	Cloudy
**Protein (g/l)**	16	26	44
**Specific weight**	1.016	1.022	1.032
**Cell count (10 × 10^9^/l)**	4.50	2.70	13.03
**Conclusion**	Hygroma or seroma	Hygroma or seroma and blood	Hygroma or seroma and mild inflammation

**Table 2:** Results of different filtering strategies.

Strategy ^1^	Purebred Piètrain			Crossbred	10 Unrelated Controls	No. of Variants with Different Predicted Impact			after Filtering against Validation Cohort		
	Case 1	Dam/Sire of Case 1	Full-sib Of Case 1	Case 3		High	Moderate	Low	High	Moderate	Low
Piètrain-specific simple AR	1/1	0/1	0/1 or 0/0	0/0	0/0	1	33	37	0	2	5
Piètrain-specific compound heterozygous AR	0/1	0/1 or 0/0	0/1 or 0/0	0/0	0/0	0	4	9	0	1	1
Piètrain-specific XR	1/-	0/1/0/-	0/-	0/0	0/0	0	2	5	0	2	5
Piètrain-specific AD (de novo)	0/1	0/0	0/0	0/0	0/0	0	6	7	0	3	3
Case 3-specific AR/AD	0/0	0/0	0/0	0/1 or 1/1	0/0	50	872	1663	12	189	299

^1^ AR denotes autosomal recessive, XR denotes X-linked recessive, and AD denotes autosomal dominant modes of inheritance.

## Data Availability

All data generated or analyzed during this study are available from the corresponding author on reasonable request.

## Supplementary Materials

The following are available online at https://www.mdpi.com/2073-4425/12/2/207/s1, Figure S1: Runs of homozygosity across the autosomes of 24 analyzed Piètrain pigs, including two cases (red lines) and 22 controls (blue lines). Figure S2: Average coverage over 300 kb sliding windows as determined by whole-genome sequencing (WGS) of the analyzed Piètrain family and the unrelated case 3; note that the red lines indicate an individual’s average coverage over the whole genome. Table S1: Sample designations and breed information of the whole-genome sequenced pigs. Table S2: Genomic regions of interest identified by homozygosity and linkage analyses in the Piètrain family. Table S3: Haplotype analysis for three segments on chromosome X. Table S4: Genomic variants detected in the WGS of the Piètrain case 1 based on different filtering strategies. Table S5: Genomic variants detected in the WGS of the unrelated case 3. Video S1: Video illustrating the clinical phenotype of case 2. Video S2: Video illustrating the clinical phenotype of case 3.
