# Supplementary figures and images for: Phenotypic and Genomic Analysis of Cystic Hygroma in Pigs
Source: Genes (Basel). 2021 Jan 31;12(2):207. doi: 10.3390/genes12020207 (PMC7911466; doi:10.3390/genes12020207)

Supplementary Figure S1

CHR1

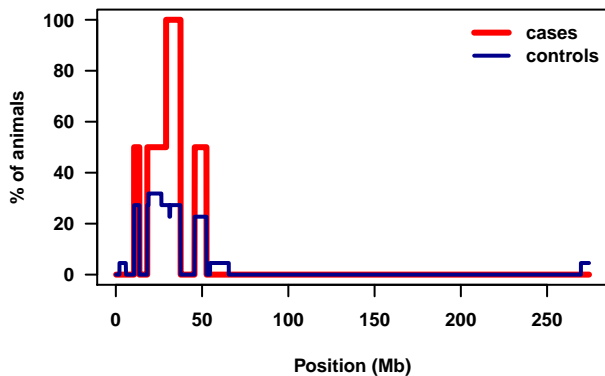

CHR2

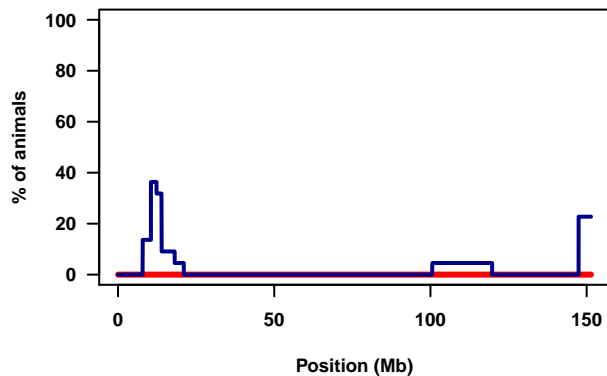

CHR3

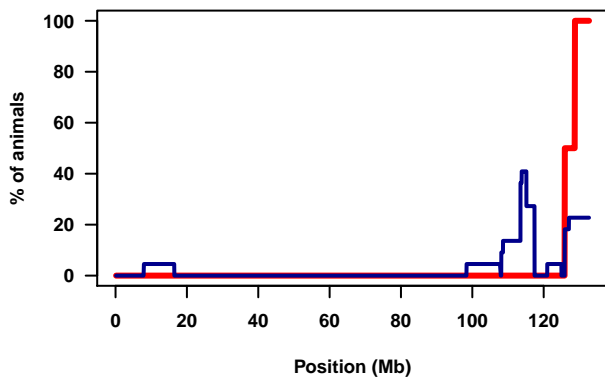

CHR4

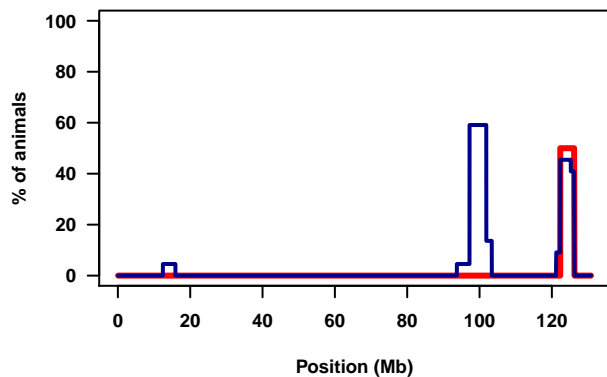

CHR5

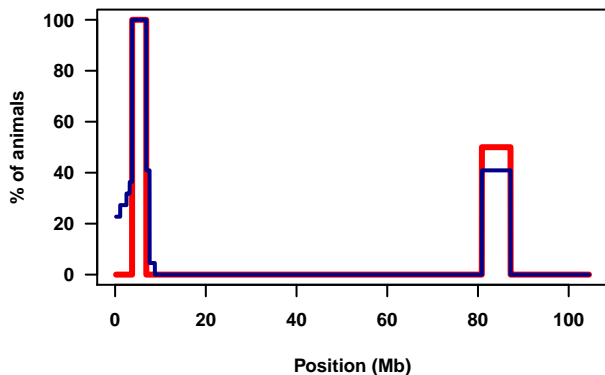

CHR6

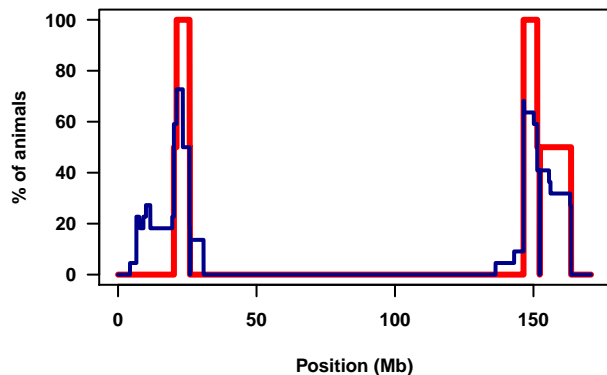

CHR7

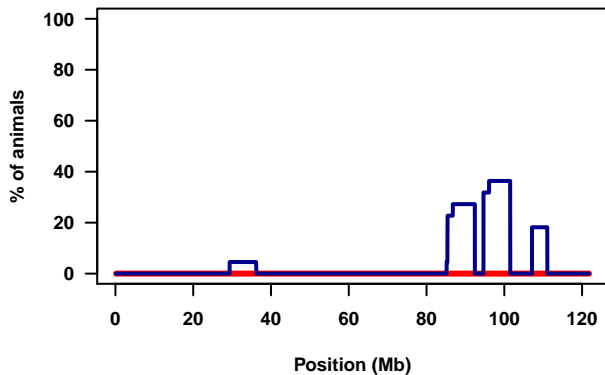

CHR8

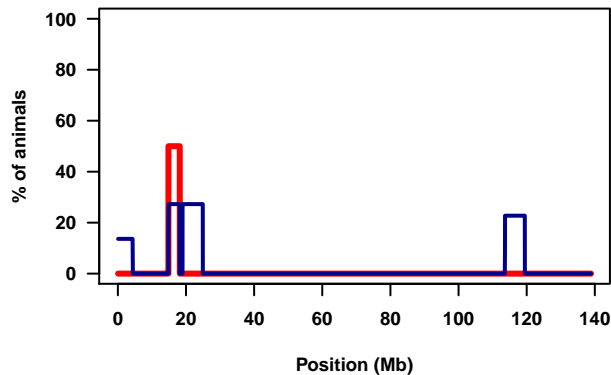

CHR9

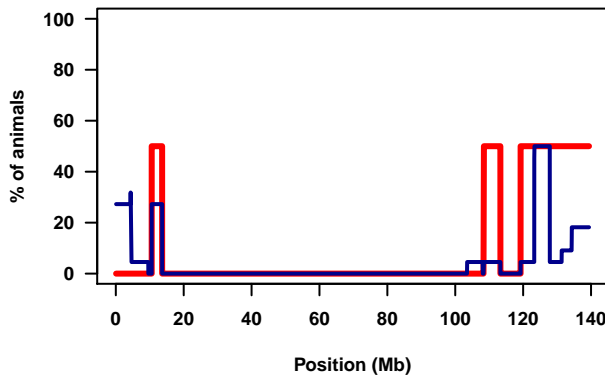

CHR10

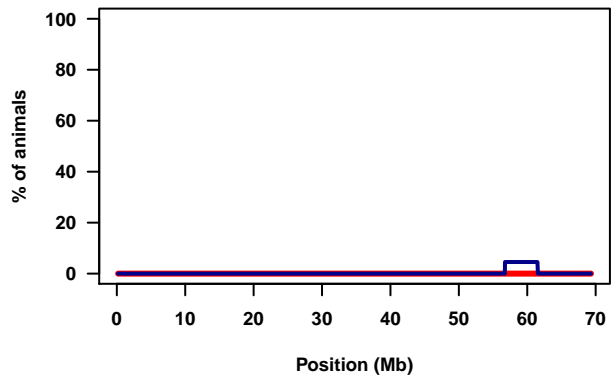

CHR11

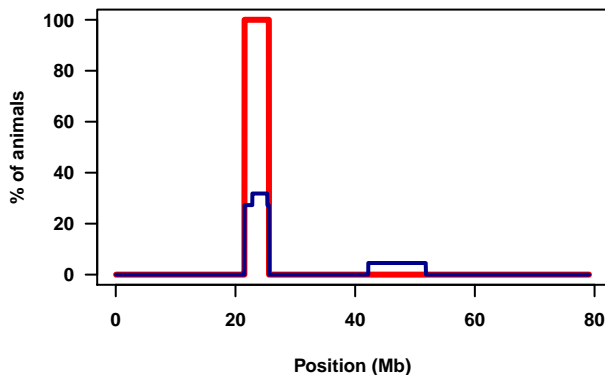

CHR12

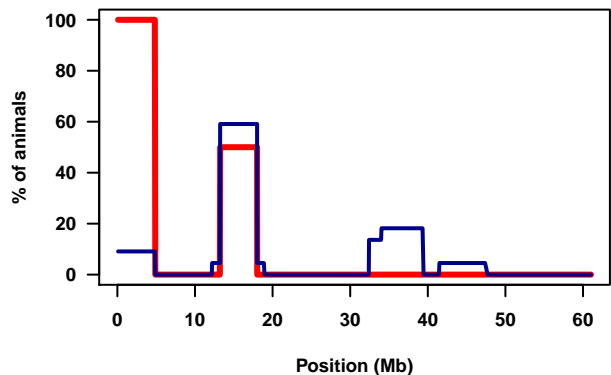

**CHR13**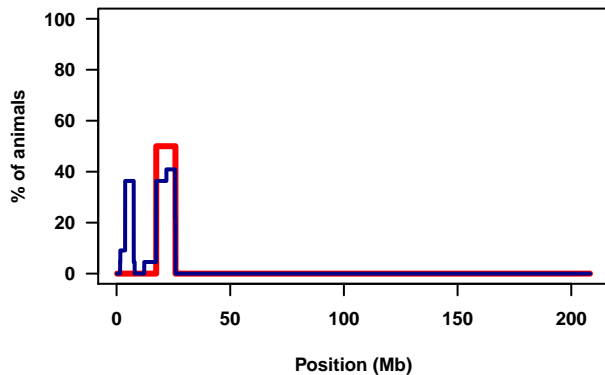**CHR14**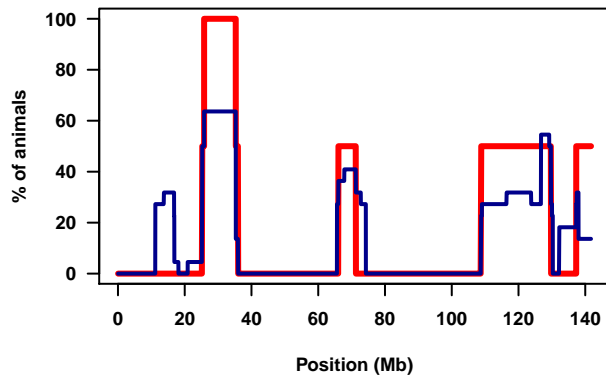**CHR15**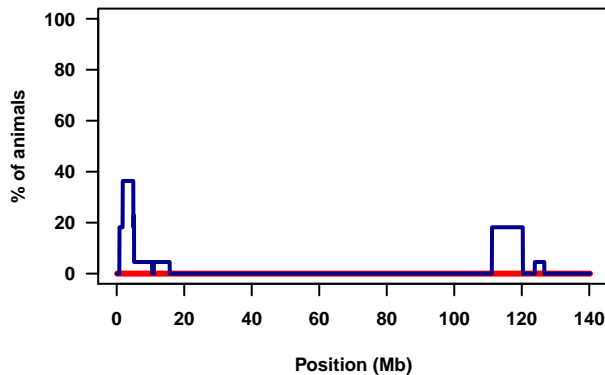**CHR16**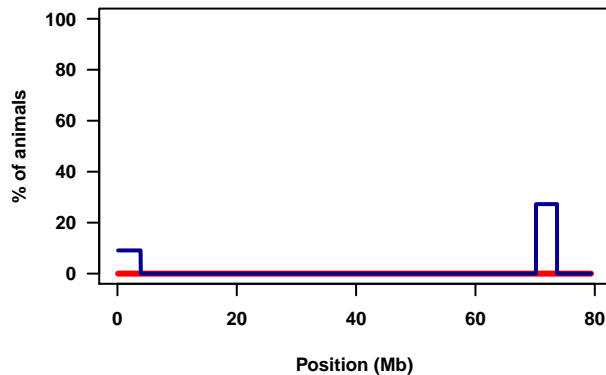**CHR17**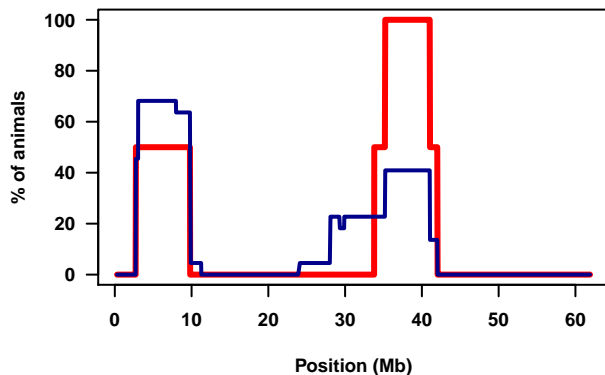**CHR18**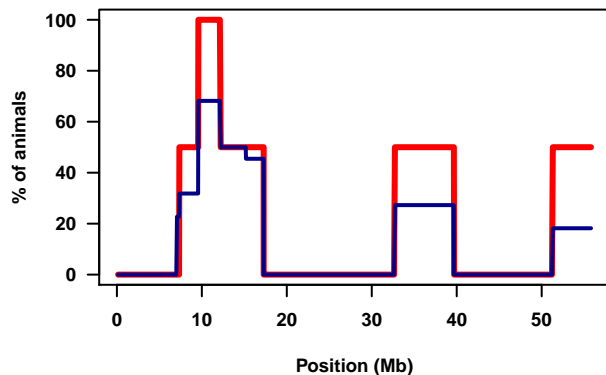

Supplement: Supplementary file 1 [file genes-12-00207-s001.zip › supplementary/Suppl_FigureS1_homozygous_regions.pdf]

## Supplementary Figure S2

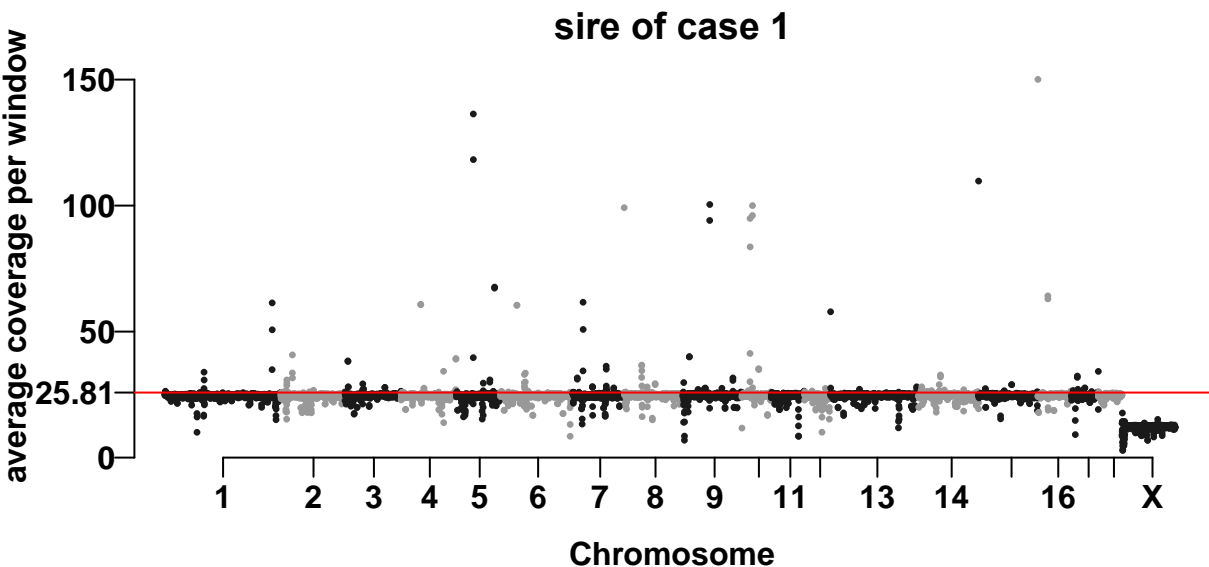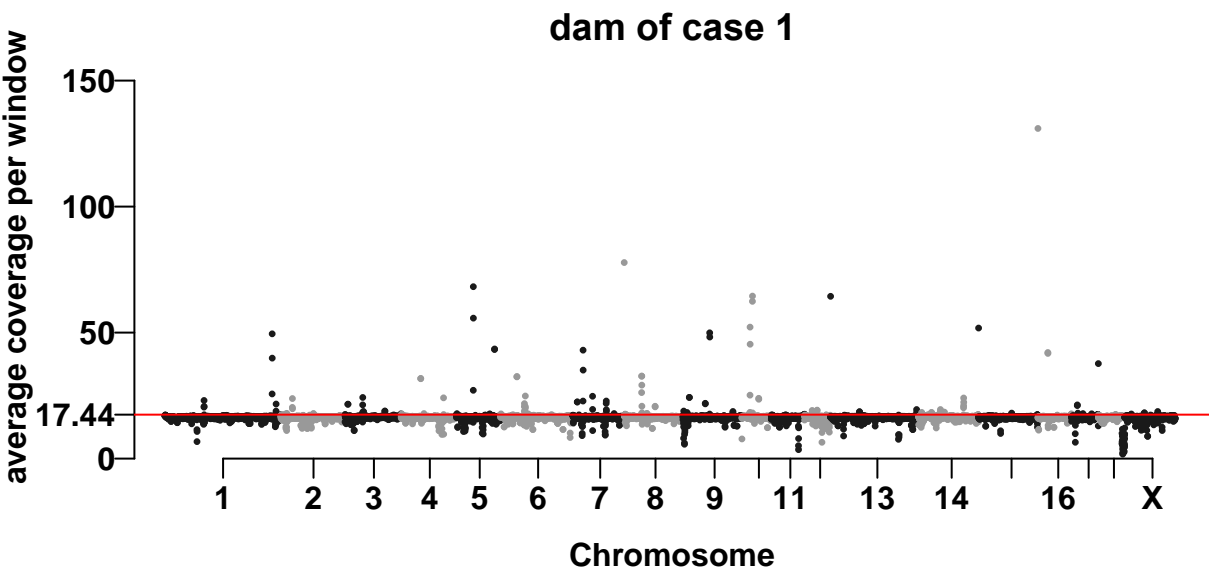

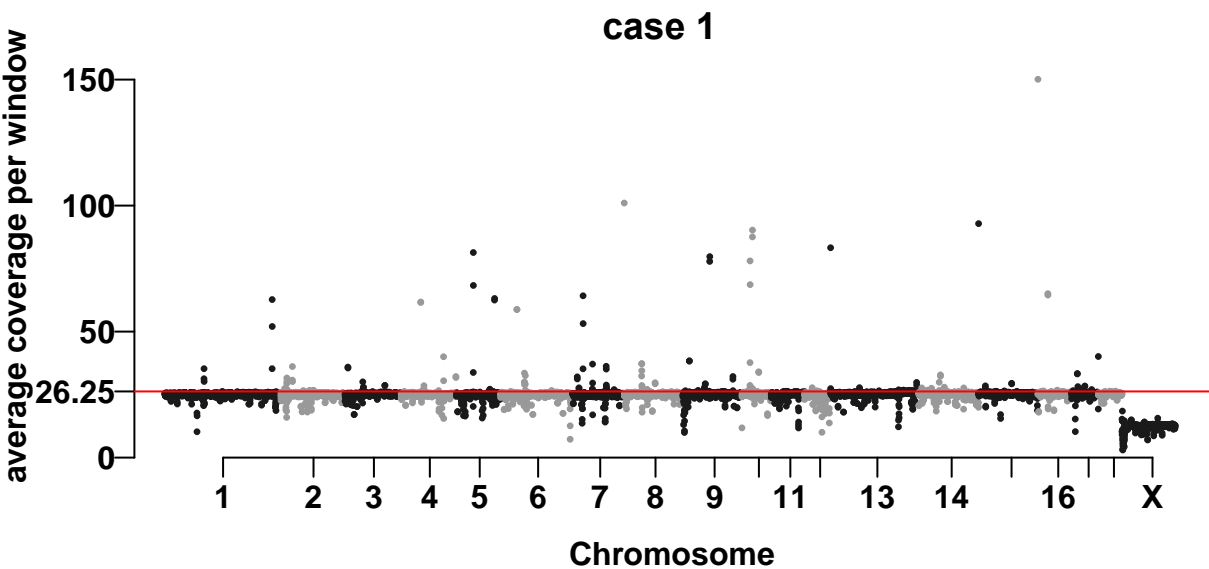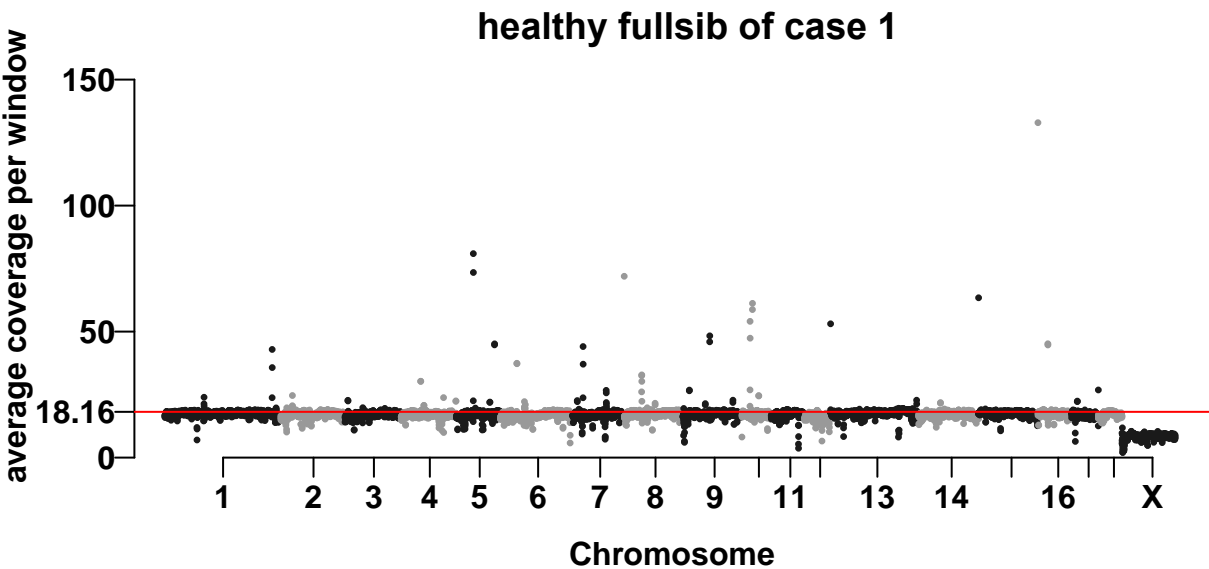

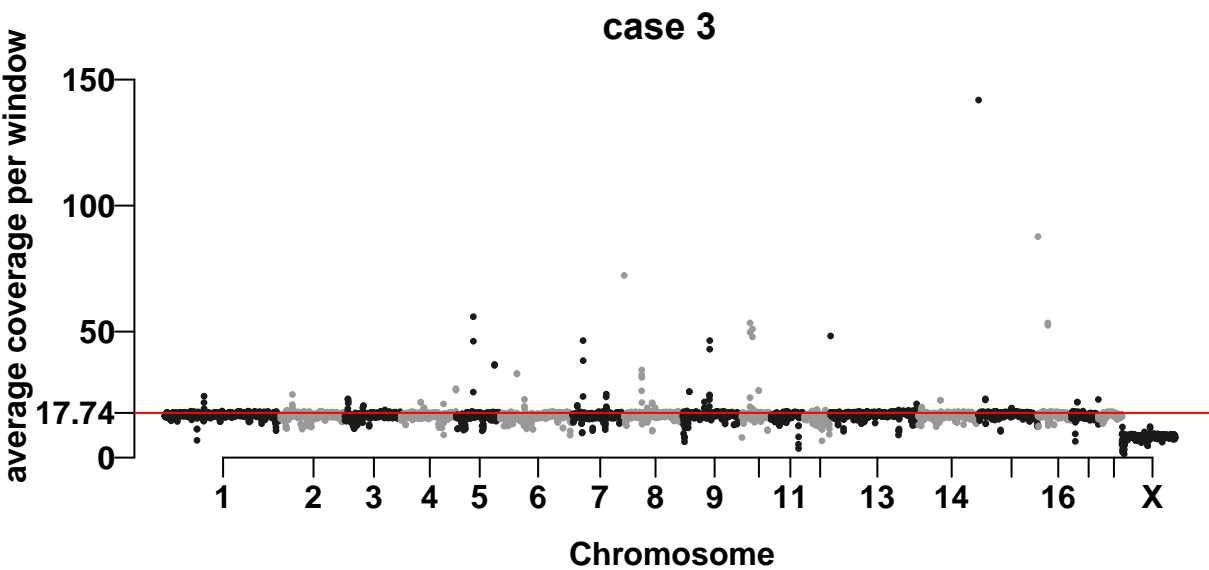

Supplement: Supplementary file 1 [file genes-12-00207-s001.zip › supplementary/Suppl_FigureS2_coverage.pdf]
